# Eatwell Guide: modelling the dietary and cost implications of incorporating new sugar and fibre guidelines

**DOI:** 10.1136/bmjopen-2016-013182

**Published:** 2016-12-21

**Authors:** Peter Scarborough, Asha Kaur, Linda Cobiac, Paul Owens, Alexandr Parlesak, Kate Sweeney, Mike Rayner

**Affiliations:** 1 Nuffield Department of Population Health, British Heart Foundation Centre on Population Approaches for Non-Communicable Disease Prevention, University of Oxford, Oxford, UK; 2 Department of Public Health, Burden of Disease Epidemiology, Equity and Cost-Effectiveness Programme, University of Otago, Wellington, New Zealand; 3 Global Nutrition and Health, Institute of Nutrition and Midwifery, Metropolitan University College, Copenhagen, Denmark; 4 Public Health England, Liverpool, UK

**Keywords:** diet, non-communicable disease scenario modelling, optimisation, food price, food-based dietary guidelines, non-linear programming

## Abstract

**Objectives:**

To model food group consumption and price of diet associated with achieving UK dietary recommendations while deviating as little as possible from the current UK diet, in order to support the redevelopment of the UK food-based dietary guidelines (now called the Eatwell Guide).

**Design:**

Optimisation modelling, minimising an objective function of the difference between population mean modelled and current consumption of 125 food groups, and constraints of nutrient and food-based recommendations.

**Setting:**

The UK.

**Population:**

Adults aged 19 years and above from the National Diet and Nutrition Survey 2008–2011.

**Main outcome measures:**

Proportion of diet consisting of major foods groups and price of the optimised diet.

**Results:**

The optimised diet has an increase in consumption of ‘potatoes, bread, rice, pasta and other starchy carbohydrates’ (+69%) and ‘fruit and vegetables’ (+54%) and reductions in consumption of ‘beans, pulses, fish, eggs, meat and other proteins’ (−24%), ‘dairy and alternatives’ (−21%) and ‘foods high in fat and sugar’ (−53%). Results within food groups show considerable variety (eg, +90% for beans and pulses, −78% for red meat). The modelled diet would cost £5.99 (£5.93 to £6.05) per adult per day, very similar to the cost of the current diet: £6.02 (£5.96 to £6.08). The optimised diet would result in increased consumption of n-3 fatty acids and most micronutrients (including iron and folate), but decreased consumption of zinc and small decreases in consumption of calcium and riboflavin.

**Conclusions:**

To achieve the UK dietary recommendations would require large changes in the average diet of UK adults, including in food groups where current average consumption is well within the recommended range (eg, processed meat) or where there are no current recommendations (eg, dairy). These large changes in the diet will not lead to significant changes in the price of the diet.

**Strengths and limitations of this study**

- This paper provides the scientific rationale for the new proportions in the Eatwell Guide using optimisation modelling.
- Although optimisation modelling can identify diets that achieve recommendations with minimal changes from current consumption, it does not take human behaviour into account, so it is unclear how achievable the modelled diets are.
- Our price data are based on foods that are sold in supermarkets. We have not adjusted for the popularity of different brands.
- We also do not allow for preparing products from scratch, which may be a cheaper alternative when judged purely by economic cost but may not be when labour costs for preparation of food are considered.

## Introduction

In July 2015 the UK Scientific Advisory Committee on Nutrition (SACN) produced a report on dietary carbohydrates based on a comprehensive review of the scientific literature,^1^ which incorporated its updated views about the recommended level of dietary carbohydrates for a healthy diet. The report concluded that the level for the mean population intake of free sugars should be reduced from 11% to 5% of dietary energy (and that consumption of sugar-sweetened drinks should be minimised), and that the recommended level for the mean adult population of fibre intake should be increased from 23–24 to 30 g/day (as measured using the AOAC method).^1^ Free sugars ‘include sugars added to foods and beverages by the manufacturer, cook or consumer, and sugars naturally present in honey, syrups, fruit juices and fruit juice concentrates’.^2^

The UK government adopted the new SACN recommendations and Public Health England (PHE) was asked to redevelop the UK's food guide (the eatwell plate^3^) to incorporate the new recommendations. PHE conducted consumer research to address how the format of the food guide should be updated to increase public understanding of the food guide, which led to various changes, including changing the name of the food categories to place emphasis on products that can be considered more environmentally sustainable; inclusion of health messaging and front-of-pack nutrition labels; removal of some foods that are high in fat or sugar from the main section of the guide.^4^ The result was the Eatwell Guide, launched in March 2016,^5^ which splits the diet into five categories and provides a pie chart where the angles for each category represent the proportion of the diet that should consist of that category. The five categories are ‘fruit and vegetables’; ‘potatoes, bread, rice, pasta and other starchy carbohydrates’; ‘beans, pulses, fish, eggs, meat and other proteins’; ‘dairy and alternatives’ and ‘oils and spreads’.

In this paper, we describe the optimisation modelling that was used to calculate the angles of the new Eatwell Guide. The angles are taken from a new modelled diet scenario (reported here) where the population mean consumption of food groups are altered such that all of the UK dietary recommendations for the average adult intake of foods and nutrients are achieved, but which deviates from the currently consumed UK diet as little as possible.

Food price is a key motivator for food choices.^6^ Unhealthy diets have frequently been shown to be cheaper than healthy diets.^7^ ^8^ In this paper, we calculate the cost of attaining the diet represented by the Eatwell Guide (the ‘Eatwell Guide’ scenario) compared with the currently consumed diet. Additionally, we have modelled a scenario where the old recommendations for free sugars and fibre were met (the ‘old recommendations’ scenario), in order to estimate the impact of the changes proposed by the latest SACN report.

## Methods

### Optimisation modelling

We conducted an optimisation modelling analysis using the non-linear generalised reduced gradient (GRG) algorithm (based on the work of Lasdon, Fox and Ratner).^9^ The analyses were conducted in Excel using the Solver function. The variables in the analysis were mean consumption levels of 125 food categories, and the constraints were achieving dietary recommendations in the UK, including daily recommended values for macronutrients proposed by the fore-runner of SACN—the Committee on Medical Aspects of Food and Nutrition Policy (COMA)^10^ and SACN itself.^1^ ^11^ ^12^ The constraints are shown in table 1. The objective function was the deviation of the modelled diet from current consumption in the UK. For food category i, we calculated a deviation index as follows:1
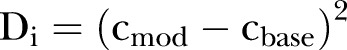
where c_mod_ is modelled consumption (in g/d) of i in the scenario and c_base_ is current consumption of i. The optimisation consisted of finding the diet that met all constraints while minimising the sum of D_i_ across all food categories. The objective function for the optimisation modelling was therefore2
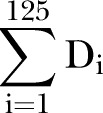
This objective function was selected following previous work that has built objective functions on the assumption that individuals facing economic constraints will choose a diet as similar as possible to their current consumption patterns.^13–15^ We used the square of the distance between the modelled and baseline consumption levels as this measure discriminates against large changes in single food categories in favour of small changes in many food categories, thereby discriminating against solutions with unrealistically large consumptions of a small number of food groups. We chose to model on the basis of quantity of food consumed in grams per day rather than kilocalories per day as evidence suggests that individuals regulate food consumption by volume as well as energy consumed.^16^ We conducted sensitivity analyses with two alternative objective functions—one where the difference in consumption in the modelled and baseline scenarios is based on kilocalories per day, and one where the difference is calculated as the absolute standardised percentage change in consumption in grams per day (an objective function previously used elsewhere^14^).

**Table 1:** Old and new recommendations used as constraints in the linear programming

	Old recommendations	Individual target or population average	Source	Eatwell Guide	Individual target or population average	Source
Nutrients						
Energy	No increase in kcal			No increase in kcal		
Carbohydrates	≥50% of food energy	Population average	1	≥50% of food energy	Population average	1
Free sugars	≤11% food energy	Population average	10	≤5% food energy	Population average	1
Fat	≤35% food energy	Population average	10	≤35% food energy	Population average	10
Saturated fat	≤11% food energy	Population average	10	≤11% food energy	Population average	10
Protein	≥14.5 and ≤15.5% of energy			≥14.5 and ≤15.5% of energy		
Salt	≤2363 mg sodium	Population average	11	≤6 g/2363 mg sodium	Population average	11
Fibre	≥23.5 g AOAC	Population average	10	≥30 g (AOAC)*	Population average	1
Foods						
Fruits and vegetables†	≥5 portions a day	Population average	49	≥5 portions a day	Population average	49
Fish	≥2 portions a week (2×20 g a day), one of which should be oily	Individual target	12	≥2 portions a week (2×20 g a day), one of which should be oily	Individual target	12
Red and processed meat	≤70 g/day	Individual target	50	≤70 g/day	Individual target	50

*Equivalent 18 g non-starch polysaccharide fibre.

†Includes a maximum of: 1 portion of juice (from fruit juice or that in a smoothie); 1 portion beans. Portion sizes: 30 g dried fruit; 150 mL fruit juice; smoothies assumed to contain 50% juice; 80 g all other fruits and vegetables.

NB. AOAC, Association of Official Analytical Chemists method for total dietary fibre analysis.

We modelled two sets of dietary constraints: the ‘Eatwell Guide’ scenario includes all of the current UK dietary recommendations; the ‘old recommendations’ scenario includes the recommendations for free sugars and fibre before they were updated by the recent SACN report. To assess the relative stringency of the constraints, we conducted sensitivity analyses where each of the constraints was relaxed by 1% in turn and recorded the value of the objective function for each analysis.^15^ The further the deviation from the value of the objective function in the primary analysis, the more difficult it was for the optimisation to meet the constraint.

We used data collected for the National Diet and Nutrition Survey (NDNS) between 2008 and 2011^17^ to calculate current mean adult intake of foods in the UK. We removed all data collected on children and adolescents under the age of 19, and applied the NDNS survey weights to our analyses to account for differential response rate by age and sex. We included data on all participants that collected food diary data for at least 3 days. Our sample included 1491 adults; of which, 841 were women.

The NDNS collected data by food diaries over 4 days. The foods that were recorded in the food diaries were matched with *food items* from over 8000 foods in the UK Nutrient Databank food composition table,^18^ and portion size estimates were made using a list of standard portion sizes^19^ but also household measures, pack sizes and photos. The NDNS categorises foods by allocating each *food item* into one of the 140 *subfood groups,* which in turn are categorised into 58 *food groups.* For our analyses, we did not include data on vitamin and mineral supplements, alcoholic drinks (NB: this explains the small difference in kilocalories consumption from our analyses and those reported in the NDNS), artificial sweeteners and infant or baby food, which left us with 125 *subfood groups.* In order to model the food-based recommendations shown in table 1 and to combine the results of the optimisation modelling into categories used by the Eatwell Guide, it was necessary to supplement the UK Nutrient Databank with estimates of the proportion of each *food item* that consisted of foods that are used for the constraints (table 1) or are included in the Eatwell Guide categories. This was performed by applying the following methods to all *food items* from the 125 *subfood groups* included in our analysis:

1. For non-composite foods, the classification system used for the eatwell plate (the predecessor of the Eatwell Guide)^20^ was applied, which is adopted equivalently in the Eatwell Guide.
2. For commonly consumed composite products, that is, for lasagne, spaghetti bolognese, cottage pie, meat pies, fruit pies, pizza and soup, the information from the homemade recipe versions of these products in the NDNS was used to derive an approximate allocation of ingredient to Eatwell Guide categories.
3. For commonly consumed foods and condiments that are not allocated to a group in the Eatwell Guide that is, for mayonnaise (full and low fat), salad cream, ketchup, chips, roast potatoes and custard online recipes were used to derive approximate allocation to Eatwell Guide categories.

**Figure 1:**
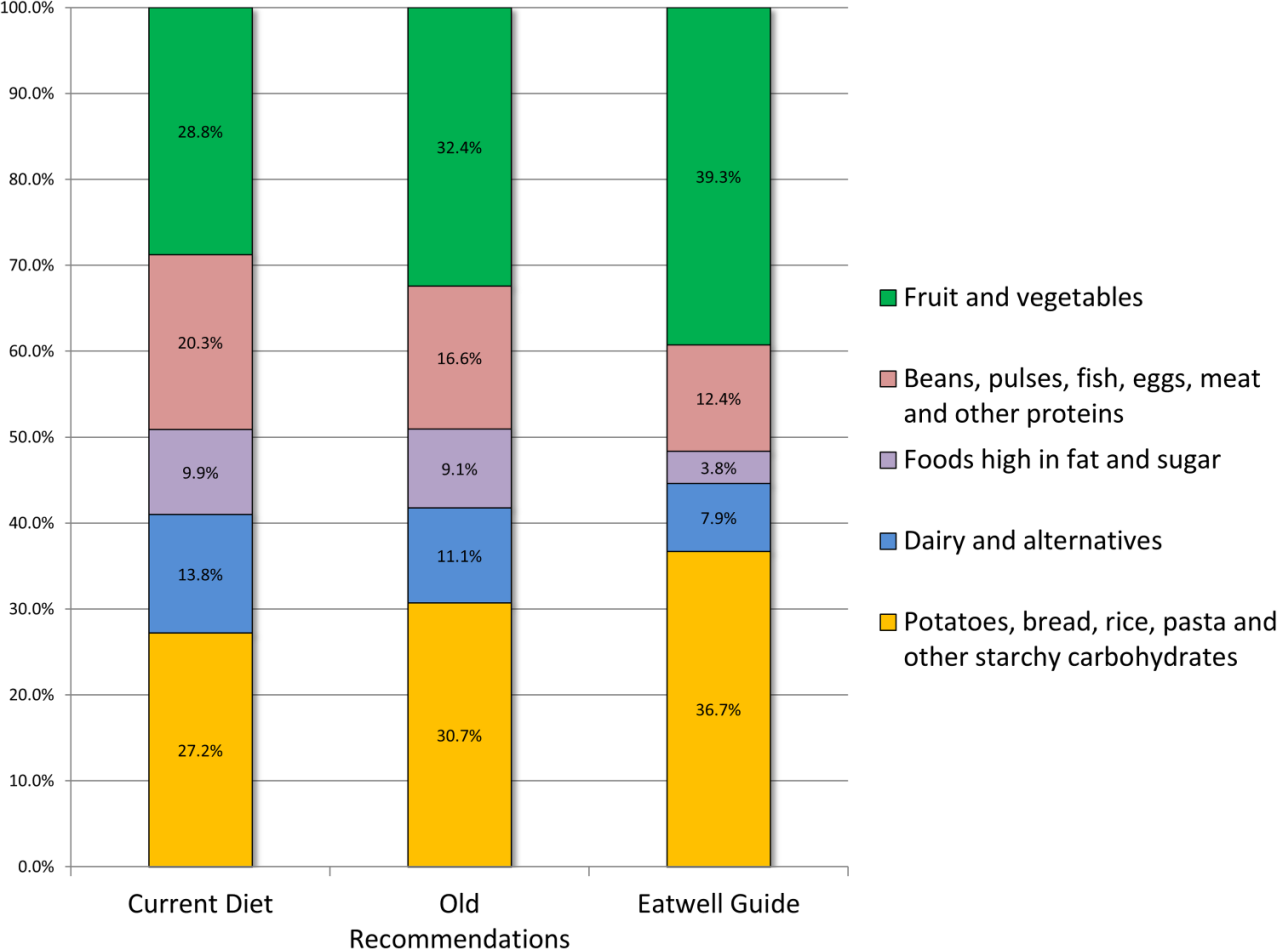
Breakdown of the diet by Eatwell Guide categories for current consumption, the ‘Eatwell Guide’ scenario and the ‘old recommendations’ scenario. NB: In the published Eatwell Guide, the foods high in fat and sugar category was replaced with ‘Oils and spreads’, and some of the foods were removed from the plate with the message ‘Eat less often and in small amounts’.

Following the same method used for designing the eatwell plate,^20^ we halved the weight of beverages (but only for the calculation of their contribution to the percentages of diets by Eatwell Guide categories shown in figure 1. For results reported in table 3 we included the full weight of beverages.)

Note that after consumer research, the names of four of the eatwell plate food categories were changed in order to emphasise sustainable choices within those categories.^4^ In this paper, we use the new Eatwell Guide names of these food categories. For the remaining category (‘foods high in fat and sugar’), we use the older name from the eatwell plate. This is because the Eatwell Guide uses the name ‘oils and spreads’ and moves many of the foods from this category to the bottom left-hand corner of the guide.^5^ However, our analyses include these removed foods so we retain the older (more descriptive) name. For clarity, the angle of the ‘oils and spreads’ segment of the published Eatwell Guide is 1% and represents merely the oils and spread of ‘foods high in fat and sugar’. A full description of the definitions used for food categorisation is provided in online supplementary appendix 1.

10.1136/bmjopen-2016-013182.supp1 supplementary appendix

For each NDNS participant, we averaged across *food items* to estimate the mean consumption (in g/day), mean nutritional quality (g per 100 g for macronutrients and micronutrients) and mean contribution to SACNs food-based recommendations and Eatwell Guide categories by *subfood group.* We then averaged across participants (weighted by survey weights) to estimate the mean values of these variables in our data set. This gave us a data set of 125 *subfood groups* with mean consumption for consumers and non-consumers combined, and mean nutritional quality and contribution to food-based recommendations and Eatwell Guide categories of consumed foods within the *subfood groups.* This was the data set we used for the optimisation modelling.

### Price of diets

In order to put a price on the currently consumed diet and the modelled scenarios, we estimated the price (£ per kg) of each of the 125 *subfood groups* in the optimisation data set. To do this, we collected 14 638 prices of 7575 food items on sale in 8 different UK online supermarkets in March 2016 using automated data collection techniques. Inclusion criteria for the foods were: being part of the 125 NDNS *subfood groups*, being indicated in the NDNS list of examples for these groups,^17^ and being included in the UK Nutrient Databank food composition table.^18^ Each of these foods was assigned to a single *subfood group* in the optimisation data set, and the mean price and SD of each *subfood group* was calculated. The SDs were combined linearly across *subfood groups* to produce estimates of SD, SE and CIs around price estimates. There was a wide variation in the number of foods with price data for each *subfood group* due to variations in the availability of products in the eight supermarkets. Only one food was found for the ‘ready meals based on bacon and ham’ *subfood group,* whereas 305 foods were found for the ‘crisps and savoury snacks’ *subfood group*.

The NDNS measures weight of food as consumed, whereas the price data are taken from food as sold, so we applied two transformations to convert the price data from £ per kg sold to £ per kg consumed. First, we used category-specific estimates from the WRAP food waste report 2012^21^ to adjust for unavoidable waste. Second, we used category-specific conversion factors from raw to cooked weight from the McCance and Widdowson series of food composition tables.^22^ Some of the *subfood groups* are explicitly homemade (eg, ‘Biscuits, homemade’)—for these *subfood groups* we assigned the price of equivalent foods that can be purchased readymade from supermarkets. The homemade *subfood groups* contributed to 12.5% (by weight) of all consumed *subfood groups*.

## Results

### Optimisation modelling

Figure 1 shows the breakdown by Eatwell Guide food categories of the currently consumed diet, the ‘Eatwell Guide’ scenario and the ‘old recommendations’ scenario. In order to meet the latest UK dietary recommendations, modelled consumption of ‘potatoes, bread, rice, pasta and other starchy carbohydrates’ and ‘fruit and vegetables’ increased from 27.2% to 36.7% (by weight) for the former and from 28.8% to 39.3% for the latter, with corresponding falls in the modelled proportion of food consumed from all other categories. Much of this change has been prompted by the changes in recommendations for free sugars and fibre—under the ‘old recommendations’, dietary change from current consumption in all categories was much smaller: the biggest change was a 3.7 percentage-point decrease in ‘beans, pulses, fish, eggs, meat and other proteins’ from 20.3% to 16.6%.

Table 2 shows the nutrient composition of the currently consumed diet and the two dietary scenarios. Many of the nutritional changes are directly mandated by the constraints of the optimisation (shown in table 1), for example, modelled consumption of free sugars falls from 11% to 5% of dietary energy in the ‘Eatwell Guide’ scenario. The modelled consumption of n-3 fatty acids increases in both scenarios with an almost 50% increase in the ‘Eatwell Guide’ scenario. Of the 15 micronutrients considered in these analyses, 11 increase in modelled consumption in the ‘Eatwell Guide’ scenario and 8 increase in modelled consumption in the ‘old recommendations’ scenario. The four micronutrients that decrease in modelled consumption in the ‘Eatwell Guide’ scenario compared to current consumption are calcium, iodine, riboflavin and zinc. Changes in calcium and iodine modelled consumption amount to <1% of current consumption. Average consumption of zinc currently meets the recommended nutrient intake (RNI), but under the ‘Eatwell Guide’ scenario and the ‘old recommendations’, this would fall to just below the RNI.

**Table 2:** The mean intake of nutrients and fruit and vegetable portions in the UK population, and results for the ‘Eatwell Guide’ and ‘old recommendations’ scenarios

	Current mean intake*	Old recommendations	Eatwell Guide
Total energy, kcal/day	1711	1711	1711
Protein, g/day (% energy)	73 (17%)	66 (16%)	66 (16%)
Carbohydrate, g/day (% energy)	213 (47%)	244 (54%)	264 (58%)
Free sugars, g/day (% energy)	50 (11%)	50 (11%)	23 (5%)
Total sugars, g/day (% energy)	89 (20%)	94 (21%)	81 (18%)
Fat, g/d (% energy)	69 (36%)	59 (31%)	51 (27%)
Saturated fatty acids, g/day (% energy)	25 (13%)	19 (10%)	15 (8%)
AOAC fibre, g/day	18	24	30
Sodium, mg/day	2266	2225	2070
Calcium, mg/day (% RNI)	800 (114%)	771 (110%)	794 (113%)
*cis*-n-3 fatty acids, g (% DRV)	1.9 (17%)	2.2 (19%)	2.7 (24%)
Iron, mg (% RNI)	10 (99%)	11 (111%)	13 (126%)
Folate, µg (% RNI)	244 (122%)	272 (136%)	329 (165%)
Iodine, µg (% RNI)	148 (106%)	146 (105%)	147 (105%)
Potassium, mg (% RNI)	2664 (76%)	2877 (82%)	3411 (97%)
Riboflavin, mg (% RNI)	1.5 (124%)	1.4 (118%)	1.4 (120%)
Thiamin, mg (% RNI)	1.4 (161%)	1.5 (174%)	1.8 (203%)
Vitamin A (retinol equivalents), µg (% RNI)	1044 (161%)	966 (149%)	1211 (186%)
Vitamin B12, mg/day (% RNI)	5.4 (362%)	4.7 (313%)	5.4 (363%)
Vitamin B6, mg (% RNI)	2.1 (137%)	2.0 (136%)	2.4 (161%)
Vitamin C, mg (% RNI)	84 (209%)	93 (233%)	117 (292%)
Vitamin D, µg (% RNI)	2.9 (29%)	3.2 (32%)	4.2 (42%)
Vitamin E, mg (% EAR)	8.8 (126%)	9.3 (133%)	9.6 (137%)
Zinc, mg/day (% RNI)	8.6 (104%)	7.5 (91%)	7.7 (93%)
Fruit and vegetables, portions/day	4.2	5.0	6.9

*Weighted average for all adults (>18 years) in the National Diet and Nutrition Survey 2008–2011.

AER, average estimated requirement. All DRVs, RNIs and AERs are averages for adult population groups; DRV, daily recommended value; RNI, recommended nutrient intake.

Table 3 shows the impact of the scenarios on food category consumption. The modelled diets require large falls in red meat and processed meat consumption. In the ‘Eatwell Guide’ scenario, red meat and processed meat are reduced by over 75% compared to current consumption but balanced with an increased modelled consumption of beans, pulses and other legumes by over 85%. Modelled consumption from the dairy and alternatives category falls by 21% in the ‘Eatwell Guide’ scenario, due to large falls in consumption of cheese and yoghurt (modelled consumption of milk falls by only 9% in the ‘Eatwell Guide’ scenario). Under the ‘old recommendations’, there was no need to reduce sugar-sweetened beverage (SSB) modelled consumption because the current consumption of free sugars is ∼11% of dietary energy, which is the same as the old recommendation. However, under the ‘Eatwell Guide’ scenario, mean SSB modelled consumption more than halves, from 120 to 59 mL/day. The increased fibre recommendation prompts huge increases in the ‘potatoes, bread, rice, pasta and other starchy carbohydrates’ category, with modelled consumption of wholemeal bread and high fibre breakfast cereals more than doubling compared to current consumption levels. In contrast, the ‘old recommendations’ prompt increases in these food groups by only 45% and 40%, respectively.

**Table 3:** The mean* price and intake of selected food groups in the UK population, and results for the ‘Eatwell Guide’ and ‘old recommendations’ scenarios

	Current mean diet g/day*, £/day (SE)†	Old Recommendations g/day, £/day (SE)†	Eatwell Guide g/day, £/day (SE)†
Fruit and vegetables‡	342, £1.24 (0.01)	400, £1.42 (0.01)	526, £2.08 (0.02)
Fruit§	102, £0.50 (0.08)	115, £0.57 (0.09)	205, £1.05 (0.12)
Fruit juice	63, £0.22 (0.02)	64, £0.22 (0.02)	32, £0.11 (0.02)
Dried fruit	4.6, £0.04 (0.04)	5.6, £0.04 (0.04)	7.9, £0.06 (0.05)
Vegetables¶	171, £0.58 (0.09)	212, £0.66 (0.09)	284, £0.91 (0.10)
Potatoes, bread, rice, pasta and other starchy carbohydrates‡	281, £0.73 (0.01)	335, £0.89 (0.01)	473, £1.18 (0.01)
Brown and wholemeal bread	33, £0.06 (0.01)	48, £0.09 (0.01)	83, £0.15 (0.02)
White bread	49, £0.10 (0.02)	51, £0.10 (0.02)	68, £0.14 (0.02)
Rice	27, £0.05 (0.01)	28, £0.05 (0.01)	28, £0.05 (0.01)
Pasta	25, £0.09 (0.02)	27, £0.09 (0.02)	35, £0.12 (0.02)
Potatoes**	91, £0.18 (0.03)	105, £0.21 (0.03)	173, £0.33 (0.04)
Breakfast cereals, high fibre	20, £0.10 (0.02)	28, £0.13 (0.03)	52, £0.25 (0.04)
Breakfast cereals, not high fibre	5.6, £0.03 (0.01)	8.1, £0.04 (0.01)	5.1, £0.03 (0.01)
Dairy and alternatives‡	221, £0.40 (0.00)	197, £0.27 (0.00)	173, £0.20 (0.00)
Milk††	170, £0.15 (0.01)	163, £0.14 (0.01)	155, £0.13 (0.01)
Cheese	17, £0.14 (0.03)	4.2, £0.03 (0.01)	2.6, £0.02 (0.01)
Yoghurt‡‡	27, £0.09 (0.01)	26, £0.09 (0.01)	12, £0.04 (0.01)
Beans, pulses, fish, eggs, meat and other proteins‡	212, £2.13 (0.02)	184, £1.67 (0.02)	160, £1.44 (0.02)
Red meat§§	35, £0.54 (0.09)	13, £0.21 (0.06)	7.7, £0.08 (0.03)
Processed meat	33, £0.36 (0.04)	17, £0.17 (0.03)	7.2, £0.06 (0.01)
White meat¶¶	35, £0.33 (0.08)	24, £0.22 (0.06)	5.0, £0.05 (0.04)
Oily fish	8.7, £0.10 (0.04)	20, £0.23 (0.07)	38, £0.47 (0.10)
Whitefish***	16, £0.23 (0.07)	20, £0.30 (0.08)	23, £0.33 (0.08)
Beans, pulses and other legumes	14, £0.03 (0.02)	25, £0.05 (0.02)	26, £0.06 (0.02)
Nuts	2.7, £0.04 (0.03)	6.1, £0.09 (0.04)	2.6, £0.03 (0.03)
Foods high in fat and sugar‡	216, £0.82 (0.01)	213, £0.87 (0.01)	103, £0.39 (0.01)
Sugar sweetened beverages	120, £0.18 (0.02)	119, £0.18 (0.02)	59, £0.09 (0.02)
Low calorie beverages	85, £0.11 (0.00)	85, £0.11 (0.00)	83, £0.11 (0.00)
Cakes, confectionary and biscuits†††	71, £0.48 (0.09)	76, £0.52 (0.09)	31, £0.19 (0.06)
Crisps and savoury snacks	6.1, £0.06 (0.03)	10, £0.11 (0.04)	6.0, £0.06 (0.03)
Oils and spreads	14, £0.07 (0.02)	4.4, £0.02 (0.01)	5.5, £0.03 (0.02)
Total price of diet, £/d (95% CIs)	**£6.02 (£5.96 to £6.08)**	**£5.81 (£5.75 to £5.87)**	**£5.99 (£5.93 to £6.05)**

NB: for complete results of each 125 subfood groups (see online supplementary material appendix 2).

NB: Prices of the food groups do not add to give the total price of the diet due to foods that do not fit into any category (eg, tea, coffee, bottled water).

*Weighted mean for all adults (>18 years) in the National Diet and Nutrition Survey 2008–2011.

†SE refers to the variance around estimates of the price of each food category *not* to the current consumption.

‡Mean consumption of Eatwell food categories (in bold) reported here are inconsistent with proportions shown in figure 1. This is because the proportions shown in figure 1 include 50% reduction of all drinks, whereas here no reduction is applied.

§‘Fruit’ includes smoothie fruit and excludes fruit juice and dried fruit.

¶‘Vegetables’ includes tomatoes, tomato puree, brassica, yellow, red and green vegetables, and other vegetables.

**‘Potatoes’ includes chips but excludes the fats used for making chips.

††‘Milk’ includes milk in cereal-based milk puddings (manufactured and homemade), coffee (made-up), semiskimmed milk, skimmed milk, whole milk and other milk.

‡‡‘Yoghurt’ does not include fromage frais and/or other dairy desserts.

§§‘Red Meat’ includes beef, lamb and pork.

¶¶‘White meat’ includes chicken and other poultry.

***‘Whitefish’ includes canned (but not fresh) tuna and shellfish.

†††‘Cakes, confectionary and biscuits’ includes biscuits, buns, cakes, pastries and fruit pies, puddings, chocolate confectionery, yogurt fromage frais and dairy desserts, sugar preserves and sweet spreads, and sugar confectionery.

SE, SEM.

10.1136/bmjopen-2016-013182.supp2 supplementary appendix

Full results of the optimisation modelling, with changes in consumption of each of the 125 *subfood groups*, is provided in online supplementary appendix 2.

### Price of diets

Table 3 also shows the changes in the price for different Eatwell Guide food categories and the whole diet under the different dietary scenarios. Achieving the ‘Eatwell Guide’ scenario has little effect on the price of the diet, which changes from £6.02 (95% CIs: £5.96 to £6.08) per adult per day in the current diet to £5.99 (£5.93 to £6.05) in the ‘Eatwell Guide’ scenario. Large increases in the price of ‘fruit and vegetables’—£0.84 (£0.79 to £0.88)—are balanced by falls in the price of ‘beans, pulses, fish, eggs, meat and other proteins’—£0.69 (−£0.75 to −£0.63). Inclusion of the updated sugar and fibre recommendations increase the price of only the recommended diet by 3%. Using previous recommendations for sugar and fibre, the modelled cost of the diet that achieved dietary recommendations was £5.81 (£5.75 to £5.87) per adult per day.

### Sensitivity analyses

Our results were sensitive to the choice of the objective function for the optimisation modelling. When the difference in baseline and modelled consumption was measured by kcal/day rather than g/day, the resultant angles of the Eatwell Guide for the ‘fruit and vegetables’, ‘beans, pulses, fish, eggs, meat and alternatives’, ‘foods high in fat and sugar’, ‘dairy and alternatives’ and ‘potatoes, bread, rice, pasta and other starchy carbohydrates’ categories were 53%, 12%, 5%, 6% and 24%, respectively. When the objective function was the absolute percentage difference in consumption, these angles were 50%, 12%, 5%, 7% and 26%, respectively. Our analysis of the stringency of the constraints showed that the final model was not affected by small changes in the carbohydrate, fat, saturated fat, fruit and vegetable, fish and meat constraints, but were heavily affected (and to a similar extent) by small changes to the free sugar, fibre and salt constraints indicating that they were the most difficult to achieve.

## Discussion

To achieve the diet recommended by the Eatwell Guide will require large changes to the average diet of UK adults, including in food groups where current average consumption is already well within the recommended range (eg, red meat and processed meat) or where there are no current recommendations (eg, dairy and alternatives). The ‘Eatwell Guide’ scenario would lead to improved nutritional quality of the diet and is likely to lead to substantial health benefits in the UK.^23^ The price of healthy diets has previously been shown to be an important barrier to their uptake,^7^ ^24^ but our analyses suggest that the changes in diet needed to achieve the Eatwell Guide do not necessarily need to result in increases in price.

The dietary shifts that are needed, including large increases in dietary carbohydrates and decreases in fat, are considerable and largely unprecedented in recent UK history. The various incarnations of the Living Cost and Food Survey^25^ have tracked food purchases since 1970s and shown that fruit and vegetable purchases increased by 30% between 1974 and 2007, and has been in decline since; meat and meat product purchases have shown small fluctuations since 1974 and are currently 7% lower; whereas milk and cheese sales have fallen by 34%.^25^ In contrast, the Eatwell Guide scenario requires ‘fruit and vegetable’ consumption to increase by 54%, ‘beans, pulses, fish, eggs, meat and other proteins’ to fall by 24% and ‘dairy and alternatives’ to fall by 21% compared to current consumption. Additionally, consumption of ‘foods high in fat and sugar’ needs to fall by 53%. In order to achieve these ambitious targets, we need to learn from successful public health dietary interventions,^26–28^ implement and evaluate new and ongoing dietary interventions such as the proposed sugary drinks levy, and develop new interventions that impact on the whole diet as opposed to individual nutrients or food groups.

If the diet represented by the Eatwell Guide were to be achieved, there may be substantial environmental cobenefits. The Carbon Trust has estimated that achieving the ‘Eatwell Guide’ scenario would result in reductions in greenhouse gas emissions, water use and land use, bringing all three within globally sustainable levels.^29^ Under the Climate Change Act 2008, the Government has committed to achieving an 80% reduction from 1990 levels of carbon emissions by 2050.^30^ Animal-based products generally contribute more to emissions than plant-based products,^31–33^ and consumption of more plant-based diets are associated with lower GHG emissions in the UK.^34^ However, it is unclear whether the increase in fish consumption in the ‘Eatwell Guide’ scenario is compatible with sustainable management of marine and aquatic environments. More than three-quarters of the world's fish stocks are fully exploited, overexploited or depleted.^35^ While global aquaculture production of fish and other seafood products has grown substantially since the 1970s, particularly in Asia,^36^ there are concerns about their environmental impact. The UK has moved to regulate aquacultural practices to minimise impact on the environment (eg, to manage escapes and control of diseases), but regulation in other countries may not be as rigorous.

This modelling project has provided an indication of the scale of dietary change that would be required in order to achieve the latest UK dietary recommendations. While optimisation modelling can identify diets that achieve recommendations with minimal changes from current consumption, it does not take human behaviour into account, so it is unclear how achievable the modelled diets are. Econometric studies that have investigated the relationship between price and demand for foods and drinks, for example, show that a reduction in demand for one product results in measurable effects on the demand for complementary or substitute products. For example, a reduction in demand for meat is associated with reduced intake of complementary products such as fats and oils and increases in demand for substitute products such as fish.^37^ Optimisation modelling does not take account of these behaviours.

Our sensitivity analyses have demonstrated that the results of our analyses are sensitive to choice of the objective function. Recently what counts as ‘5 a day’ has slightly changed with respect to how much smoothies can contribute.^38^ Incorporating the new advice makes no difference to the results reported here.

The optimisation modelling conducted in this paper has been carried out at the population level. An alternative approach would be to conduct individual diet modelling,^39^ where a separate optimisation model is constructed for each of the individuals in the NDNS and the aggregated results are combined to produce an optimised population model. There are two advantages of the individual diet modelling approach. First, the final population model is based on an aggregate of results at the individual-level models and, therefore, it is possible to calculate the variance (and hence CIs) around population-level results. This would provide an assessment of the robustness of the results. A second advantage of individual-level diet modelling is that baseline diets are actual diets whereas the average population diet used as the baseline for these analyses is a composite diet that is not actually consumed by anyone in the population. However, the objective of this analysis was to construct an average diet for the UK population that meets the population goals set out in table 1. The majority of these goals are population rather than individual-level goals, that is, they are targets for the mean level of consumption within a population as opposed to targets for individual-level consumption. Using an individual-level approach would result in an average population diet where everyone in the population meets the population goals, whereas the population-level approach produces results where roughly half the population meet the population goals. Conceptually, we believe that the population-level approach is better suited to optimisation modelling for meeting population dietary goals. However, because of the advantages described above it would be useful to cross-validate these results against an individual-diet modelling approach.

Our price data are based on foods that are sold in supermarkets. We have not adjusted for the popularity of different brands; in our analyses, all available foods are treated equally regardless of sales of the foods. This may affect our estimate of the price of the diets, although a comprehensive analysis of a linked data set of purchases and prices (eg, the Living Cost and Food Survey^25^) would be needed to evaluate which direction (if any) this may bias our results. We also do not allow for preparing products from scratch, which may be a cheaper alternative when judged purely by economic cost but may not be when labour costs for preparation of food are considered.^40^ However, our main interest was in the difference between the price of the currently consumed diet and the scenarios, where the proportion of homemade dishes did not substantially change. Therefore, this limitation is unlikely to affect our conclusions.

Our results on micronutrient quality of the diet do not account for differences in the bioavailability of nutrients consumed from different foods. For example, it has been estimated that bioavailability of iron in a mixed diet is ∼14–18% but only 5–12% for vegetarian diets with no iron stores.^41^ Reductions in the bioavailability of this magnitude could impact on the nutritional adequacy of the diet for population subgroups. Further work with individual diet modelling could explore this possibility further.

The NDNS which provided the data for these analyses is subject to under-reporting,^17^ which explains why the energy levels of the baseline and modelled diets are fairly low. It is likely that baseline levels of specific foods are also under-reported, which could exaggerate or underplay the changes in diet that are needed to meet the constraints depending on whether the constraints encourage greater consumption (eg, fruit and vegetables) or less consumption (eg, salt consumption). This will have less of an impact on macronutrient constraints that are set as a percentage of total energy, unless there is differential under-reporting by nutrient. The reductions in mean sodium intake in our modelled diets result from the constraint to reduce mean salt consumption to 6 g/day (table 1) and may mean that some individual diets fall well below this level. Whether this would lead to adverse health consequences for some people is a subject of debate.^42^ Estimates of salt consumption come from the NDNS food intake data and, therefore, do not include salt that is added during cooking or at the table.

We find that the ‘Eatwell Guide’ scenario would have a similar cost to the current average diet in the UK. A recent systematic review compared the cost of healthy and unhealthy diets.^6^ The review identified 14 studies that compared costs based on food group-level changes in the diet and found that the healthy diets were $1.48 ($1.01, $1.95) more expensive than less healthy diets. When adjusted for kilocalories consumption (as our analyses are), this price difference increased slightly to $1.54. Our lack of price increase for the ‘Eatwell Guide’ scenario is, therefore, not generally supported by the literature. This difference is likely to be because the ‘Eatwell Guide’ scenario is a modelled diet rather than a real healthy diet that is achieved by a subgroup of the population. What our analyses show is that it is *possible* to achieve the UK dietary recommendations without increasing the cost of the average UK diet. This finding is supported by a pilot study in Australia which found that recommended healthy diets can be cheaper than currently consumed diets^43^ although Lee *et al* included takeaway sales and alcohol in their measures of currently consumed diets.

Previous studies have applied optimisation modelling to identify diets that meet nutritional recommendations. Minimising the difference from currently consumed diets from a small sample of adults in the United States, Masset *et al*^44^ found that achieving dietary recommendations issued by the World Cancer Research Fund and the American Institute of Cancer Research required substantial increases in fruit and vegetable consumption and reductions in meat and eggs. However, in contrast to our results, they found that achieving the dietary recommendations required an increase in the consumption of dairy products and a reduction in the consumption of whole and refined grains. A similar study using a French population^45^ found that achieving dietary recommendations while minimising differences to currently consumed diets required increases of fruit and vegetables by 62%, increases in starches and grains by 37% and reductions in meat, fish, poultry and eggs by 12%. In contrast to our study, they found that an increase in dairy products by 19% was required. Two optimisation modelling studies from the UK^46^ and New Zealand^47^ included greenhouse gas emission reductions as constraints alongside nutritional constraints and found that it is possible to achieve a diet that meets dietary recommendations and reduces emissions, but these diets require a reduction in consumption of meat and dairy and an increase in consumption of plant-based foods, consistent with the recommendations of the Eatwell Guide. These comparisons are limited as the studies were conducted using different populations with differences in baseline diets and the set of nutritional constraints used in the modelling. With the exception of salt, all of the constraints used in our modelling were based on macronutrients or foods, whereas the other modelling exercises included many more micronutrient recommendations. Additionally, the French study used individual diet modelling, the studies used different objective function (our sensitivity analyses show that our results are sensitive to the choice of objective function) and used ‘acceptability constraints’ where consumption of individual food groups was constrained to high levels of consumption observed in the baseline data set. Our choice of objective function mitigated against the need for acceptability constraints—in our main results, modelled consumption of each of the 125 food groups was never higher than 25% of the maximum consumption observed in the NDNS data set.

The dietary scenarios that were modelled here resulted in some decreases in micronutrients (although only mean consumption of zinc moved to lower than the recommended intake). Future work should model the potential impact of achieving the Eatwell Guide on distributions of micronutrient consumption in population subgroups, and assess the potential impact of reduced average meat and dairy consumption on micronutrient requirements. This could be conducted with individual diet modelling that would contribute to the development of appropriate meal plans (such as those developed by the British Nutrition Foundation^48^). Future work could also investigate the difference in price of diets by different subgroups of the population, and could estimate the likely health impact of achieving the ‘Eatwell Guide’ scenario.
