# Supplementary material for: Eatwell Guide: modelling the dietary and cost implications of incorporating new sugar and fibre guidelines
Source: BMJ Open. 2016 Dec 21;6(12):e013182. doi: 10.1136/bmjopen-2016-013182 (PMC5223664; doi:10.1136/bmjopen-2016-013182)
Supplement: supplementary appendix [file bmjopen-2016-013182supp_appendix1.pdf]

Supplementary material: Definitions and methods for converting recommendations into constraints used for 'The Eatwell Guide: modelling the dietary and cost implications of incorporating new sugar and fibre guidelines'

Definitions

Fruit and vegetables

The definition of a fruit or a vegetable for the analyses presented here was taken from the Government's NHS Choices website which has details about which foods count towards meeting the 5-a-day recommendation[1].

Fish

The definitions for fish and oily fish were taken from the UK Scientific Advisory Committee on Nutrition (SACN) report on the benefits and risks of fish consumption[2]. Fish and oily fish included all species listed below.

Table1: Oily fish and white fish categorisation

Oily fish		White fish	
Anchovies	Sprats	Ayr	Monk fish
Bloater	Swordfish	Cat fish	Parrot fish
Cacha	Trevally*	Cod	Plaice
Carp	Trout	Coley	Pollack
Eel	Tuna (fresh)	Dab*	Pomfret
Herring	Whitebait	Dover sole	Red & grey mullet
Hilsa		Flounder	Red fish
Horse mackerel*		Flying fish	Red Snapper
Jack fish		Gurnard*	Rock Salmon/Dogfish
Katla		Haddock	Rohu
Kipper		Hake	Sea bass
Mackerel		Halibut	Sea bream
Orange roughy		Hoki	Shark
Pangas		John dory	Skate
Pilchards		Kalabasu	Tilapia
Salmon		Lemon sole	Tuna (tinned)*
Sardines		Ling	Turbot
Scad*		Marlin*	Whiting

* Included in this list but not in the original table from the SACN report

[1] NHS choices. 5 A Day: what counts? Available from: http://www.nhs.uk/Livewell/5ADAY/Pages/Whatcounts.aspx
[2] SACN (2004) Advice on fish consumption: benefits and risks. London: TSSO https://www.food.gov.uk/sites/default/files/cot/fishreport2004full.pdf

Red and processed meat

The definition for red and processed meat was adapted from information included in Table A45 of the SACN report on iron and health[3]: Red and processed meat has therefore been considered to be:

(I) Carcass meats and offal from red meat animals i.e. cow, sheep, pig, deer, hare and goat but not white meat animals i.e. chicken, turkey, goose, duck, pheasant, guinea fowl, partridge, pigeon and rabbit.

(ii) Sliced preserved meat from red meat animals (e.g. ham).

(ii) The meat (including offal) component of the following products made with at least some meat (including offal) from red meat animals: haggis, black pudding, brawn, faggots, meat balls, beef paste, Cornish pasties, pork pies, sausage rolls, scotch eggs and all other foods containing some meat (including offal) from red meat animals.

(iv) The whole of the following products made with at least some meat (including offal) from red meat animals: sausages, salami, frankfurters, corned beef, spam, pate, meat loaf, burgers and similar.

The following products are examples of foods not considered to contain any meat: bacon flavour crisps, pork scratching, lard, beef dripping, Bovril, gravy, gelatine.

Converting recommendations into constraints for linear programming

Fruit and vegetables (general)

Current Government advice is that the recommended daily diet should consist of at least five portions of fruit and vegetables[1].

One portion of fruit or vegetables (including pulses and beans) was considered to be 80g (as eaten, edible portion, drained (if canned) except if the fruit or vegetable :

a) was dried and then it was considered to be 30g; or

b) had been converted to a juice or smoothie and then it was considered to be 75g[4] [5]. (NB: the weight of all beverages in the dataset was initially divided by 2 to compensate for their high water content.)

[3] Table A45, p336 of SACN (2010) Iron and health. London: TSSO. Available from: https://www.gov.uk/government/uploads/system/uploads/attachment_data/file/339309/SACN_Iron_and_Health_Report.pdf

Fruit or vegetable that was part of a composite food was considered to contribute to the daily recommendation. For the analyses presented here the total number of portions of fruit and vegetable was calculated to include partial portions found in composite foods.

Fruit and vegetable juice, smoothies, pulses and beans (specific)

Current Government advice is that only:

a) one portion of fruit or vegetable juice (75g)

b) two portions of smoothie (2x 75g) and

c) one portion of pulses or beans (80g);

contribute towards meeting the 5-a-day recommendations[1].

Red and processed meat

Current Government advice is for adults with a high intake of red and processed meat (over 90g per day) to reduce their daily average intake to 70g per day[6]. The simplest way of converting this to a constraint was to suggest that the recommended diet must contain no more than 70g per day of red and processed meat.

Fish and of oily fish

Current Government advice is that the population should consume, on average, at least two portions (2 X 140g) of fish a week, one of which should be oily[2]. The simplest way of converting this to a constraint was to suggest that the recommended diet should contain at least 40g per day of fish of which 20g should be oily.

---

[4] NHS Choices 5aday portion guide. Available from: http://www.nhs.uk/livewell/5aday/documents/downloads/5aday_portion_guide.pdf

[5] Gatenby S, Hunt P, Rayner M (1995). The National Food Guide: development of dietetic criteria and nutritional characteristics. Journal of Human Nutrition and Dietetics 1995,8:323-334.

[6] SACN (2010) Iron and health. London: TSSO. Available from: https://www.gov.uk/government/uploads/system/uploads/attachment_data/file/339309/SACN_Iron_and_Health_Report.pdf
