# Supplementary material for: Eatwell Guide: modelling the dietary and cost implications of incorporating new sugar and fibre guidelines
Source: BMJ Open. 2016 Dec 21;6(12):e013182. doi: 10.1136/bmjopen-2016-013182 (PMC5223664; doi:10.1136/bmjopen-2016-013182)
Supplement: supplementary appendix [file bmjopen-2016-013182supp_appendix2.pdf]

Appendix 2: Amount consumed (in grams) by NDNS sub-food group.

Main food group code	Main food group description	Sub-food group code	Sub-food group description	Current average diet (g/person/d)	Old recommendations (g/person/d)	Eatwell Guide (g/person/d)
1	PASTA RICE AND OTHER CEREALS	1C	PIZZA	11.8	12.5	11.3
1	PASTA RICE AND OTHER CEREALS	1D	PASTA MANUFACTURED PRODUCTS & READY MEALS	5.9	6.1	5.5
1	PASTA RICE AND OTHER CEREALS	1E	OTHER PASTA INCLUDING HOMEMADE DISHES	19.3	21.0	29.7
1	PASTA RICE AND OTHER CEREALS	1F	RICE MANUFACTURED PRODUCTS & READY MEALS	2.4	3.3	2.4
1	PASTA RICE AND OTHER CEREALS	1G	OTHER RICE INCLUDING HOMEMADE DISHES	24.7	24.4	25.6
1	PASTA RICE AND OTHER CEREALS	1R	OTHER CEREALS	8.3	11.5	10.7
2	WHITE BREAD	2R	WHITE BREAD (NOT HIGH FIBRE; NOT MULTISEED BREAD)	49.5	50.9	67.6
3	WHOLEMEAL BREAD	3R	WHOLEMEAL BREAD	18.3	27.7	53.7
4	OTHER BREAD	4R	OTHER BREAD	2.9	8.1	3.4
5	HIGH FIBRE BREAKFAST CEREALS	5R	WHOLEGRAIN & HIGH FIBRE BR'FAST CEREALS	19.4	26.8	49.7
6	OTHER BREAKFAST CEREALS	6R	OTHER BREAKFAST CEREALS (NOT HIGH FIBRE)	5.6	8.1	5.1
7	BISCUITS	7A	BISCUITS MANUFACTURED / RETAIL	12.3	16.3	6.3
7	BISCUITS	7B	BISCUITS HOMEMADE	0.1	0.1	0.1
8	BUNS CAKES PASTRIES & FRUIT PIES	8B	FRUIT PIES MANUFACTURED	1.3	3.3	1.3
8	BUNS CAKES PASTRIES & FRUIT PIES	8C	FRUIT PIES HOMEMADE	0.4	0.5	0.4
8	BUNS CAKES PASTRIES & FRUIT PIES	8D	BUNS CAKES & PASTRIES MANUFACTURED	13.1	14.1	3.1
8	BUNS CAKES PASTRIES & FRUIT PIES	8E	BUNS CAKES & PASTRIES HOMEMADE	3.1	4.0	2.4
9	PUDDINGS	9C	CEREAL BASED MILK PUDDINGS - MANUFACTURED	5.6	4.3	4.7
9	PUDDINGS	9D	CEREAL BASED MILK PUDDINGS - HOMEMADE	0.6	0.4	0.5
9	PUDDINGS	9E	SPONGE PUDDINGS - MANUFACTURED	0.3	0.3	0.3
9	PUDDINGS	9F	SPONGE PUDDINGS - HOMEMADE	0.6	0.6	0.6
9	PUDDINGS	9G	OTHER CEREAL BASED PUDDINGS - MANUFACTURED	2.2	3.8	2.1
9	PUDDINGS	9H	OTHER CEREAL BASED PUDDINGS - HOMEMADE	2.9	3.1	2.6
10	WHOLE MILK	10R	WHOLE MILK	31.3	29.8	13.7
11	SEMI SKIMMED MILK	11R	SEMI SKIMMED MILK	100.5	99.1	111.4
12	SKIMMED MILK	12R	SKIMMED MILK	19.9	18.6	15.5

Main food group code	Main food group description	Sub-food group code	Sub-food group description	Current average diet (g/person/d)	Old recommendations (g/person/d)	Eatwell Guide (g/person/d)
13	OTHER MILK AND CREAM	13B	CREAM (INCLUDING IMITATION CREAM)	2.8	0.5	1.9
13	OTHER MILK AND CREAM	13R	OTHER MILK	10.2	8.8	6.9
14	CHEESE	14A	COTTAGE CHEESE	0.5	0.2	0.5
14	CHEESE	14B	CHEDDAR CHEESE	3.2	0.0	1.2
14	CHEESE	14R	OTHER CHEESE	12.6	3.0	0.0
15	YOGURT FROMAGE FRAIS AND DAIRY DESSERTS	15B	YOGURT	27.3	26.2	12.1
15	YOGURT FROMAGE FRAIS AND DAIRY DESSERTS	15C	FROMAGE FRAIS AND DAIRY DESSERTS MANUFACTURED	1.7	0.9	1.6
15	YOGURT FROMAGE FRAIS AND DAIRY DESSERTS	15D	DAIRY DESSERTS HOMEMADE	0.2	0.2	0.2
16	EGGS AND EGG DISHES	16C	MANUFACTURED EGG PRODUCTS INCLUDING READY MEALS	2.6	1.5	2.3
16	EGGS AND EGG DISHES	16D	OTHER EGGS AND EGG DISHES INCLUDING HOMEMADE	16.1	10.9	0.0
17	BUTTER	17R	BUTTER	4.1	0.6	0.2
18	PUFA MARGARINE & OILS	18A	POLYUNSATURATED MARGARINE	0.0	0.0	0.0
18	PUFA MARGARINE & OILS	18B	POLYUNSATURATED OILS	0.4	0.2	0.4
19	LOW FAT SPREAD	19A	POLYUNSATURATED LOW FAT SPREAD	1.2	0.6	1.0
19	LOW FAT SPREAD	19R	LOW FAT SPREAD NOT POLYUNSATURATED	0.4	0.3	0.3
20	OTHER MARGARINE FATS AND OILS	20A	BLOCK MARGARINE	0.1	0.1	0.1
20	OTHER MARGARINE FATS AND OILS	20B	SOFT MARGARINE NOT POLYUNSATURATED	0.0	0.0	0.0
20	OTHER MARGARINE FATS AND OILS	20C	OTHER COOKING FATS AND OILS NOT PUFA	1.3	0.0	1.0
21	REDUCED FAT SPREAD	21A	REDUCED FAT SPREAD (POLYUNSATURATED)	1.6	0.2	1.2
21	REDUCED FAT SPREAD	21B	REDUCED FAT SPREAD (NOT POLYUNSATURATED)	5.0	2.4	1.1
22	BACON AND HAM	22A	READY MEALS / MEAL CENTRES BASED ON BACON AND HAM	0.0	0.0	0.0
22	BACON AND HAM	22B	OTHER BACON AND HAM INCLUDING HOMEMADE DISHES	14.4	5.6	0.0
23	BEEF VEAL AND DISHES	23A	MANUFACTURED BEEF PRODUCTS INCLUDING READY MEALS	5.8	5.5	5.6
23	BEEF VEAL AND DISHES	23B	OTHER BEEF & VEAL INCLUDING HOMEMADE RECIPE DISHES	20.5	11.5	0.0
24	LAMB AND DISHES	24A	MANUFACTURED LAMB PRODUCTS INCLUDING READY MEALS	1.3	1.5	1.3
24	LAMB AND DISHES	24B	OTHER LAMB INCLUDING HOMEMADE RECIPE DISHES	7.4	0.0	2.4
25	PORK AND DISHES	25A	MANUFACTURED PORK PRODUCTS INCLUDING READY MEALS	0.5	0.2	0.5
25	PORK AND DISHES	25B	OTHER PORK Â INCLUDING HOMEMADE RECIPE DISHES	7.3	0.0	2.1

Main food group code	Main food group description	Sub-food group code	Sub-food group description	Current average diet (g/person/d)	Old recommendations (g/person/d)	Eatwell Guide (g/person/d)
26	COATED CHICKEN	26A	MANUFACTURED COATED CHICKEN / TURKEY PRODUCTS	5.5	1.1	3.5
27	CHICKEN AND TURKEY DISHES	27A	MANUFACTURED CHICKEN PRODUCTS INCL READY MEALS	7.8	5.1	5.6
27	CHICKEN AND TURKEY DISHES	27B	OTHER CHICKEN / TURKEY INCL HOMEMADE RECIPE DISHES	32.7	23.8	0.0
28	LIVER & DISHES	28R	LIVER AND DISHES	1.5	0.0	1.3
29	BURGERS AND KEBABS	29R	BURGERS AND KEBABS PURCHASED	5.7	1.4	3.6
30	SAUSAGES	30A	READY MEALS BASED ON SAUSAGES	0.1	0.1	0.1
30	SAUSAGES	30B	OTHER SAUSAGES INCLUDING HOMEMADE DISHES	11.5	8.8	2.4
31	MEAT PIES AND PASTRIES	31A	MANUFACTURED MEAT PIES AND PASTRIES	7.4	7.7	5.5
31	MEAT PIES AND PASTRIES	31B	HOMEMADE MEAT PIES AND PASTRIES	1.6	0.0	1.5
32	OTHER MEAT AND MEAT PRODUCTS	32A	OTHER MEAT PRODUCTS MANUFACTURED Â INCL READY MEALS	3.3	0.0	1.9
32	OTHER MEAT AND MEAT PRODUCTS	32B	OTHER MEAT Â INCLUDING HOMEMADE RECIPE DISHES	1.9	0.0	1.6
33	WHITE FISH COATED OR FRIED	33R	WHITE FISH COATED OR FRIED	9.3	11.0	14.1
34	OTHER WHITE FISH SHELLFISH & FISH DISHES	34C	MANUFACTURED WHITE FISH PRODUCTS INCL READY MEALS	0.8	1.2	0.8
34	OTHER WHITE FISH SHELLFISH & FISH DISHES	34D	OTHER WHITE FISH INCLUDING HOMEMADE DISHES	5.2	6.8	8.1
34	OTHER WHITE FISH SHELLFISH & FISH DISHES	34E	MANUFACTURED SHELLFISH PRODUCTS INCL READY MEALS	0.6	0.7	0.7
34	OTHER WHITE FISH SHELLFISH & FISH DISHES	34F	OTHER SHELLFISH INCLUDING HOMEMADE DISHES	3.3	5.8	4.2
34	OTHER WHITE FISH SHELLFISH & FISH DISHES	34G	MANUFACTURED CANNED TUNA PRODUCTS INCL READY MEALS	2.8	2.3	3.4
34	OTHER WHITE FISH SHELLFISH & FISH DISHES	34H	OTHER CANNED TUNA INCLUDING HOMEMADE DISHES	1.7	1.5	1.9
35	OILY FISH	35A	MANUFACTURED OILY FISH PRODUCTS INCL READY MEALS	3.6	8.9	9.2
35	OILY FISH	35B	OTHER OILY FISH INCLUDING HOMEMADE DISHES	6.4	14.3	32.3
36	SALAD AND OTHER RAW VEGETABLES	36A	CARROTS RAW	2.1	8.0	2.6
36	SALAD AND OTHER RAW VEGETABLES	36B	SALAD AND OTHER RAW VEGETABLES	23.9	26.5	46.2
36	SALAD AND OTHER RAW VEGETABLES	36C	TOMATOES RAW	18.5	20.2	30.0
37	VEGETABLES NOT RAW	37A	PEAS NOT RAW	9.0	16.8	18.4
37	VEGETABLES NOT RAW	37B	GREEN BEANS NOT RAW	3.6	11.3	5.1
37	VEGETABLES NOT RAW	37C	BAKED BEANS	15.8	23.3	35.0
37	VEGETABLES NOT RAW	37D	LEAFY GREEN VEGETABLES NOT RAW	13.7	17.4	24.9
37	VEGETABLES NOT RAW	37E	CARROTS NOT RAW	13.2	18.4	26.1

Main food group code	Main food group description	Sub-food group code	Sub-food group description	Current average diet (g/person/d)	Old recommendations (g/person/d)	Eatwell Guide (g/person/d)
37	VEGETABLES NOT RAW	37F	TOMATOES NOT RAW	12.7	13.7	16.5
37	VEGETABLES NOT RAW	37I	BEANS AND PULSES INCL READY MEAL & HOMEMADE DISHES	5.3	13.1	8.5
37	VEGETABLES NOT RAW	37K	MEAT ALTERNATIVES INCL READY MEALS & HOMEMADE DISH	2.0	4.1	2.1
37	VEGETABLES NOT RAW	37L	OTHER MANUFACTURED VEGETABLE PRODUCTS INCL RM	2.3	4.4	2.4
37	VEGETABLES NOT RAW	37M	OTHER VEGETABLES INCLUDING HOMEMADE DISHES	45.8	50.0	93.6
38	CHIPS FRIED & ROAST POTATOES AND POTATO PRODUCTS	38A	CHIPS PURCHASED INCLUDING TAKEAWAY	17.5	22.5	32.7
38	CHIPS FRIED & ROAST POTATOES AND POTATO PRODUCTS	38C	OTHER MANUFACTURED POTATO PRODUCTS FRIED/BAKED	3.7	6.8	4.0
38	CHIPS FRIED & ROAST POTATOES AND POTATO PRODUCTS	38D	OTHER FRIED / ROAST POTATOES INCL HOMEMADE DISHES	19.4	22.9	33.4
39	OTHER POTATOES POTATO SALADS & DISHES	39A	OTHER POTATO PRODUCTS & Â DISHES - MANUFACTURED	1.9	3.1	2.0
39	OTHER POTATOES POTATO SALADS & DISHES	39B	OTHER POTATOES INCLUDING Â HOMEMADE DISHES	50.2	52.6	104.3
40	FRUIT	40A	APPLES AND PEARS NOT CANNED	30.5	33.4	70.4
40	FRUIT	40B	CITRUS FRUIT NOT CANNED	12.4	14.6	18.9
40	FRUIT	40C	BANANAS	24.8	26.8	48.1
40	FRUIT	40D	CANNED FRUIT IN JUICE	2.6	4.6	2.8
40	FRUIT	40E	CANNED FRUIT IN SYRUP	0.8	1.2	0.8
40	FRUIT	40R	OTHER FRUIT NOT CANNED	28.5	32.0	68.4
41	SUGARS PRESERVES AND SWEET SPREADS	41A	SUGAR	8.8	6.5	0.0
41	SUGARS PRESERVES AND SWEET SPREADS	41B	PRESERVES	3.6	3.3	1.4
41	SUGARS PRESERVES AND SWEET SPREADS	41R	SWEET SPREADS FILLINGS AND ICING	0.3	0.3	0.3
42	CRISPS AND SAVOURY SNACKS	42R	CRISPS AND SAVOURY SNACKS	6.1	10.2	6.0
43	SUGAR CONFECTIONERY	43R	SUGAR CONFECTIONERY	1.6	2.4	1.2
44	CHOCOLATE CONFECTIONERY	44R	CHOCOLATE CONFECTIONERY	8.1	8.4	0.0
45	FRUIT JUICE	45R	FRUIT JUICE	53.1	53.0	24.4
50	MISCELLANEOUS	50A	BEVERAGES DRY WEIGHT	2.5	0.7	1.7
50	MISCELLANEOUS	50C	SOUP MANUFACTURED/ RETAIL	22.6	23.2	18.5
50	MISCELLANEOUS	50D	SOUP HOMEMADE	3.8	4.9	3.9
50	MISCELLANEOUS	50R	SAVOURY SAUCES PICKLES GRAVIES & CONDIMENTS	28.9	30.0	3.7
51	TEA COFFEE AND WATER	51A	COFFEE (MADE-UP WEIGHT)	255.9	255.9	252.4

Main food group code	Main food group description	Sub-food group code	Sub-food group description	Current average diet (g/person/d)	Old recommendations (g/person/d)	Eatwell Guide (g/person/d)
51	TEA COFFEE AND WATER	51B	TEA (MADE-UP WEIGHT)	411.8	411.8	415.6
51	TEA COFFEE AND WATER	51C	HERBAL TEA (MADE-UP WEIGHT)	22.1	22.1	22.1
51	TEA COFFEE AND WATER	51D	BOTTLED WATER STILL OR CARBONATED	80.5	80.5	80.5
51	TEA COFFEE AND WATER	51R	TAP WATER ONLY	347.3	347.3	347.3
53	ICE CREAM	53R	ICE CREAM	5.4	3.7	2.9
56	NUTS AND SEEDS	56R	NUTS AND SEEDS	2.9	7.8	3.0
57	SOFT DRINKS NOT LOW CALORIE	57A	SOFT DRINKS NOT LOW CALORIE CONCENTRATED	33.9	33.8	17.4
57	SOFT DRINKS NOT LOW CALORIE	57B	SOFT DRINKS NOT LOW CALORIE CARBONATED	68.1	67.9	31.6
57	SOFT DRINKS NOT LOW CALORIE	57C	SOFT DRINKS NOT LOW CALORIE RTD STILL	17.9	17.7	10.1
58	SOFT DRINKS LOW CALORIE	58A	SOFT DRINKS LOW CALORIE CONCENTRATED	36.1	36.1	35.0
58	SOFT DRINKS LOW CALORIE	58B	SOFT DRINKS LOW CALORIE CARBONATED	46.8	46.8	46.6
58	SOFT DRINKS LOW CALORIE	58C	SOFT DRINKS LOW CALORIE RTD STILL	1.7	1.7	1.7
59	BROWN GRANARY AND WHEATGERM BREAD	59R	BROWN GRANARY AND WHEATGERM BREAD	14.5	20.7	29.1
60	1% Fat Milk	60R	1% Fat Milk	1.6	0.9	1.6
61	SMOOTHIES 100% FRUIT AND/OR JUICE	61R	SMOOTHIES 100% FRUIT AND/OR JUICE	0.8	1.1	0.8
